# Proinflammatory cytokines regulate epidermal stem cells in wound epithelialization

**DOI:** 10.1186/s13287-020-01755-y

**Published:** 2020-06-11

**Authors:** Tong Xiao, Zhu Yan, Shengxiang Xiao, Yumin Xia

**Affiliations:** Department of Dermatology, The Second Affiliated Hospital of Xi’an Jiaotong University, 157 Xiwu Road, Xi’an, 710004 China

**Keywords:** Epidermal stem cell, Epithelialization, Proinflammatory cytokine, Skin, Wound healing

## Abstract

The skin, which serves as the first barrier of the human body, is particularly susceptible to exogenous injuries. Skin wounds, including acute burns and chronic non-healing ulcers, are commonly observed in clinics. Healing of skin wounds is a complex process, consisting of infiltration of inflammatory cells, cellular proliferation, and tissue remodeling phases, which restore the integrity and functions of the skin. Epithelialization is involved in wound healing through re-establishing an intact keratinocyte layer. Epidermal stem cells are indispensable for epithelialization, and they are regulated by multiple proinflammatory cytokines or growth factors. In this review, we summarize recent advances in the effect of these cytokines on migration, proliferation, and differentiation processes of epidermal stem cells. We also introduce promising therapeutic strategies targeting epidermal stem cells or related proinflammatory cytokines for patients with skin wounds.

## Introduction

The skin, being the outermost layer of the whole body, is susceptible to injuries and undergoes wound healing frequently. Acute wounds, especially serious burn wounds, as well as chronic wounds in elderly patients with diabetes, obesity, or vascular diseases who have impaired capacity for skin regeneration, require more effective therapies. Wound healing is a complex process consisting of the following three overlapping stages: inflammation, cell proliferation, and tissue remodeling [1]. Inflammation occurs immediately and it begins with hemostasis. During the inflammatory phase, the wound is sealed by fibrin which acts as a temporary matrix. Circulating immune cells, including neutrophils, macrophages, monocytes, mast cells, and regulatory T cells, invade the new matrix, remove the dead tissue, and control infection [2]. Cell proliferation replenishes the wound subsequently. Fibroblasts are recruited, and they secrete collagen to form granulation tissue, where angiogenesis occurs and makes it possible to transport fluid, oxygen, nutrients, and immune-competent cells [3]. Epithelialization occurs from robust activation, migration, and proliferation of epidermal stem cells to re-establish an intact keratinocyte layer [4]. Finally, restructuring of the extracellular matrix occurs during the remodeling phase, and it may lead to scar formation [5]. Stem cells (SCs) are characterized by their potential for self-renewal and differentiation into other cell types [6]. Cutaneous SCs play an essential role in wound healing, mostly based on their ability to repair cellular substrates and to enhance the migration of fibroblasts and keratinocytes, angiogenesis, and collagen and elastin production [7].

Proinflammatory cytokines are among the first factors to be produced in response to skin wounds, and they regulate the functions of immune cells in epithelialization. Proinflammatory cytokines, mainly including tumor necrosis factor (TNF), interleukin (IL)-1, IL-6, and IL-17, participate in the inflammation phase of wound healing through activating downstream cascades [8]. They also contribute to the epithelialization phase by mobilizing resident stem/progenitor cells and promoting cell proliferation and differentiation [9]. However, immune responses in wound healing are a double-edged sword. Moderate immune responses promote wound healing as normal levels of proinflammatory cytokines prevent infection and accelerate normal wound healing. Excessive production of proinflammatory cytokines is detrimental, and it possibly results in deregulated activation and differentiation of epidermal SCs, which can be observed in systemic autoimmune and metabolic disorders [10]. For example, phenotype transition from proinflammatory M1 macrophages to reparative M2 macrophages plays an important role in the switching of the inflammatory phase to the proliferation phase. M1 macrophages secrete proinflammatory cytokines, such as IL-1, IL-6, and TNF-α, as well as chemokines to recruit additional leukocytes. In contrast, anti-inflammatory cytokines, such as IL-4 and IL-13, lead to M2 macrophage subset formation, which regulate inflammation by expressing mediators as IL-1 receptor antagonist, decoy IL-1 receptor type II, and IL-10, as well as several growth factors to promote fibroblast proliferation, extracellular matrix synthesis, and angiogenesis [11–13]. The transition from M1 to M2 subset can be amplified by IL-4, and the increased number of M2 macrophages can then lead to elevation of IL-10, transforming growth factor-β (TGF-β), and IL-12 [12]. Severe inflammation has also been associated with excessive scarring. However, the exact mechanisms underlying the regulation of SCs in wound healing remain unclear. Here, we review the effect of proinflammatory cytokines on epidermal SCs in wound epithelialization and suggest novel therapeutic strategies.

## Epithelialization in skin wound involves complex inflammatory responses

Epithelialization in the proliferation phase is an essential process of wound healing, and it serves as a defining parameter of wound closure. Healing of skin wounds cannot be considered in the absence of epithelialization. Initiation, maintenance, and completion of epithelialization involve numerous factors. For example, insufficient blood supply (ischemia), infection, residual necrotic material, inadequate inflammatory or immune responses, or radiation injury may hamper the processes of epithelialization [3]. Intrinsic signals are activated in the epidermis and adjacent tissues, and they are modulated by multiple factors, including cytokines or growth factors, cellular receptors, matrix metalloproteinases (MMPs), and extracellular matrix components [14]. Complex interactions and crosstalk between keratinocytes, fibroblasts, inflammatory cells, and epidermal SCs are critical for wound closure [15].

Epithelialization commences as keratinocytes and epidermal SCs proliferate over a fibrin/fibronectin-rich provisional extracellular matrix. Both basal and supra-basal keratinocytes migrate to cover the wound area following a spatial pattern. Basal keratinocytes, including interfollicular epidermal SCs (iSCs), transient amplifying cells, and non-stem daughter cells of asymmetric proliferation, differentiate rapidly into epidermal keratinocytes. De-differentiation of terminally differentiated epidermal cells is also important in epithelialization [16]. Besides, epidermal SCs from appendages exhibit plasticity and potential for multilineage differentiation. These cells migrate from the bulges and serve as a transient bandage that allows iSCs from the interfollicular epidermis and other SCs from the upper isthmus/infundibulum to reside longer during wound healing [17]. These populations of SCs participate in epithelialization in differential ways.

TNF-α is an essential factor regulating wound healing since patients treated with TNF-α inhibitors systematically manifest delayed skin regeneration and chronic TNF-α overexpression negatively affects skin regeneration [18]. During the inflammatory phase of wound healing, TNF-α induces the synthesis of cell surface adhesion molecules on neutrophils and endothelial cells, which are important for neutrophil migration and adhesion to the endothelium. During the proliferative phase, TNF-α promotes the proliferation of keratinocytes and their expression of intracellular adhesion molecule-1 [19]. TNF-like weak inducer of apoptosis (TWEAK), a member of TNF super family, binds to its receptor fibroblast growth factor-inducible 14 (Fn14). TWEAK/Fn14 signaling modulates cutaneous inflammatory responses via regulating the cell cycle and cytokine secretion of keratinocytes as well as recruiting inflammatory cells to wound regions [20]. Topical application of recombinant TWEAK strengthens inflammatory cell infiltration and growth factor production, and it increases extracellular matrix components in wound areas [21].

Besides, other proinflammatory cytokines contribute to wound healing through recruiting immune cells and promoting the proliferation and migration of keratinocytes and fibroblasts. IL-1 produced by keratinocytes, neutrophils, and macrophages is essential for preventing wound infection. IL-1 further induces fibroblasts to secrete keratinocyte growth factor, fibroblast growth factor-7 (FGF-7), IL-6, granulocyte-macrophage colony-stimulating factor (GM-CSF), and hepatocyte growth factor [22]. These bidirectional interactions between keratinocytes and fibroblasts create a paracrine loop in the wound healing process. Inadequate IL-1 production may delay the epithelialization transition of skin lesions [18]. IL-6 exhibits both mitogenic and proliferative effect on keratinocytes during wound healing. Deficiency of IL-6 reduces neutrophil and macrophage infiltration and inhibits keratinocyte proliferation [18]. Alternatively, epidermal growth factor (EGF) and TGF-α are produced by activated macrophages and serve as the stimulus for epithelial proliferation [15]. Increased levels of growth factors, such as EGF, vascular endothelial growth factor (VEGF), and TGF-β, are prominent in the proliferative phase of wound healing [23]. Moreover, these factors regulate the expression of MMPs, which activate or inhibit several cytokines, improve leukocyte invasion, and create a chemotactic gradient to enhance inflammatory responses [24].

## The origin, differentiation, and regulation of epidermal stem cells

Skin SCs consists of epidermal SCs, dermal SCs, and melanocytic SCs, which constitute the skin structure. Epidermal SCs are a critical factor in skin homeostasis and wound healing. Distinct subtypes of epidermal SCs reside in the following areas: in the interfollicular epidermis (iSCs), in the hair follicles (hair follicle SCs, hSCs), and in the sebaceous glands (sebaceous gland SCs, sSCs) or sweat glands [6]. In postnatal skin, the interfollicular epidermal cells continuously proliferate and differentiate; thus, requiring continuous action of iSCs to maintain homeostasis. Hair follicles undergo cycles of regeneration, including the phases of growth (anagen), regression (catagen), and rest (telogen). Each subtype of SCs renews the corresponding tissue and also substitutes for other subtypes during wound healing. Dermal SCs reside in hair papilla, around pericytes, or elsewhere among other dermal cells, and they can differentiate into pericytes, fibroblasts, myoblasts, or chondrocytes [25]. The dermis contains tissue-derived SCs with an expression profile similar to adult mesenchymal SCs, where the exact identification remains unclear. Melanocytic SCs are undifferentiated melanocytic cells located in hair follicles and are the origin of melanocytes during each hair follicle cycle [26].

Many factors affect the migration, proliferation, and differentiation of epidermal SCs. Extrinsic factors mainly include regulators that form the niche of SCs, consisting of adjacent cells, matrix architecture, signaling molecules, physical forces, oxygen tension, and other environmental factors [27]. Proinflammatory cytokines, including TNF-α, IL-1, IL-6, and IL-17, are intrinsic factors, and they promote the migration, proliferation, and differentiation via both autocrine and paracrine ways. Intrinsic signaling pathways, such as mitogen-activated protein kinase, c-Myc, Wnt/β-catenin, Sonic hedgehog, and Notch, provide redundant backup signals for the actions of SCs [25].

## iSCs contribute to the epithelialization in skin wound

**Fig. 1:**
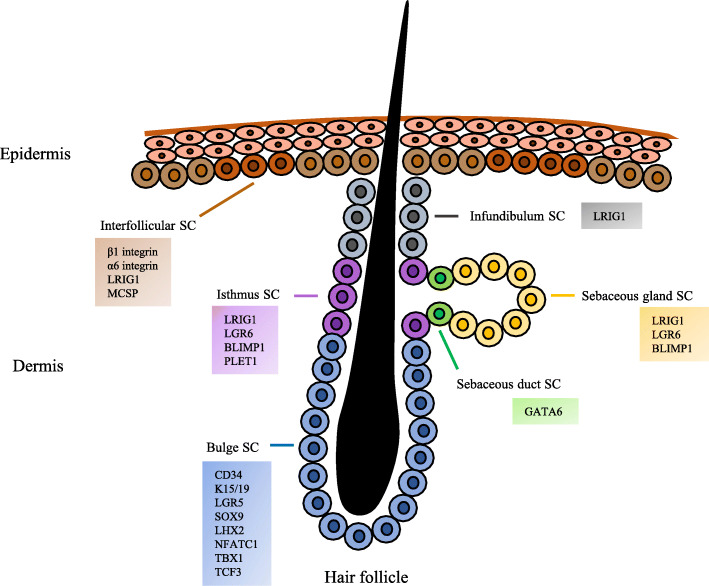
Schematic diagram of the distribution and main markers of epidermal SCs. iSCs are clustered and interspersed in the basal layer of epidermis. Most of the hair follicular SCs reside in the bulge. The isthmus SCs localize in the junction between the hair follicle and sebaceous glands. The upper part of the isthmus contains infundibular SCs. Sebaceous gland duct SCs are located at the opening of the glands while sebaceous gland SCs are located in the glands. Each population of epidermal SCs expresses distinct markers, which are shown in the colored boxes

iSCs are clustered in the basal layer of the epidermis, and they replenish the basal layer and continuously produce supra-basal cells. Recently, different markers were found to identify iSCs, including β1 and α6 integrins, Leu-rich repeats and immunoglobulin-like domains 1 (LRIG1), and melanoma-associated chondroitin sulfate proteoglycan (MCSP). Meanwhile, iSCs express low levels of transferrin receptor (CD71) and desmoglein-3. iSCs can also be traced in K14-CreER or Inv-CreER mouse strains [6, 28]. Additional lineage tracing with Dlx1-CreER and Slc1a3-CreER reporters has identified two SC populations [29]. See Fig. 1.

After detachment from the basement membrane, iSCs cease proliferation and move upwards to differentiate during epithelialization. The subtypes of SCs function depending on the thickness of the wound, or in other words, the damage status of appendages [30]. It can be concluded that the epithelialization of human partial-thickness wounds occurs primarily and rapidly by SCs in the pilosebaceous units and to a lesser extent by iSCs. In full-thickness wounds, where these adnexal structures are partly or fully destroyed, epithelialization should originate from interfollicular epidermal cells (including iSCs) at the wound margins.

When a wound-induced vacant niche exists, iSCs activate, migrate, and proliferate to occupy spatial vacancy. A switch from α6β4 to α3β1 integrin (expressed in keratinocytes) for laminin-5 (expressed on the basement membrane) binding occurs during disassembling the junctions that link keratinocytes and basal membrane [31]. Cytokines, such as IL-1, IL-6, IL-17, and TNF-α, can increase keratinocyte motility and proliferation [1]. The release of prestored IL-1 by keratinocytes is the initial signal of wound healing [22]. This autocrine fashion from keratinocytes and paracrine fashion from neutrophils, monocytes, and macrophages promote keratinocyte migration and proliferation. IL-1 induces the expressions of K6 and K16, which mark the active state of keratinocytes migrating in wounds. IL-1 also induces the gene expression of GM-CSF, TNF-α, TGF-α, and amphiregulin [4]. IL-1 plays a key role in adaptation of skin SCs to inflammatory responses via the caspase 8 signaling pathway [32]. Besides, caspase 1 and IL-1β signaling, as the downstream effector of absent in melanoma 2 (AIM2), enhances the migration of iSCs and accelerates epithelialization [33]. IL-6, mostly produced by neutrophils, has both mitogenic and proliferative effects on keratinocytes [34, 35]. IL-6 activates the signal transducer and activator of transcription (STAT)-Janus kinase (JAK) signaling pathway, allowing keratinocytes to respond to mitogenic factors that stimulate migration. By binding to its receptor IL-6Rα, IL-6 indirectly induces neutrophil and macrophage infiltration, collagen deposition, angiogenesis, and keratinocyte proliferation or migration [34, 36]. IL-17 is another potential proinflammatory cytokine that regulates keratinocytes synergistically with TNF-α, IL-1, and IL-6. IL-17A stimulates keratinocyte proliferation through the Act1-TRAF4-MEKK3-ERK5 signaling pathway [37].

TNF-α mediates keratinocyte survival and proliferation via the TNF receptor (TNFR)/nuclear factor-κB (NF-κB) signaling pathway. TNF-α regulates the secretion of cytokines in keratinocytes and cooperates with IL-1 for modulating fibroblasts. Recently, it was found that TNF-α induces AKT phosphorylation (p-AKT) in iSCs, and AKT signals activate downstream β-catenin protein [38]. Actually, TNF-α induces an epithelial-to-mesenchymal transition in cells, which initiates a fibrotic state [39]. TNF-α interacts with its receptor TNFR2 to recruit adaptor proteins and trigger signaling cascades, activating the NF-κB and activator protein (AP)-1 transcription factors, which regulate proinflammatory cytokines as well as cell survival and proliferation. TNF-α stimulates keratinocyte migration in an autocrine fashion, and it also activates fibroblasts to secrete the FGF family in a paracrine fashion [18]. In addition, the TNFR1-dependent or TNFR1-independent apoptosis affects the production of inflammatory cytokines in keratinocytes, subsequently blocking epidermal differentiation [40].

**Fig. 2:**
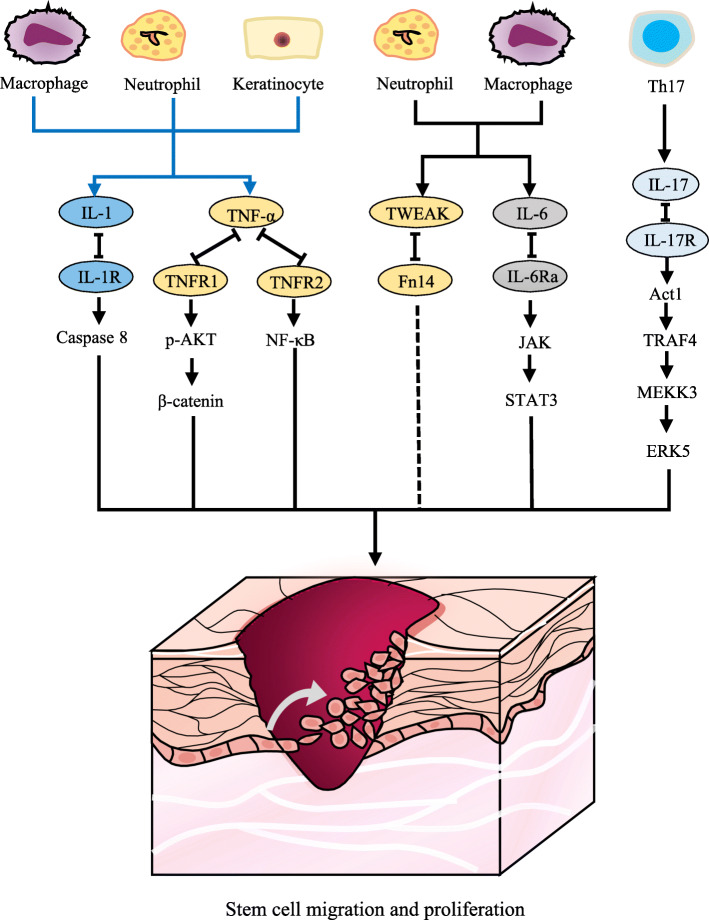
Schematic diagram of proinflammatory cytokines regulating keratinocytes or stem cells. Keratinocytes, neutrophils, and macrophages produce IL-1, which regulates stem cells through the caspase 8 signaling pathway. TNF-α binds to TNFR1 to induce AKT phosphorylation in iSCs or to TNFR2 to activate the NF-κB signaling pathway. Neutrophils and macrophages produce TWEAK, which binds to Fn14, and they have a potential effect on iSCs. IL-6 and IL-17 activate the STAT-JAK and Act1-TRAF4-MEKK3-ERK5 signaling pathways, respectively

Despite their positive effect in wound healing, excessive proinflammatory cytokines lead to failed transition from the inflammation phase to the proliferation phase, ultimately causing chronic non-healing wounds. Thus, the inhibitors of proinflammatory cytokines may be effective in the treatment of chronic wounds. The effect of proinflammatory cytokines on skin SCs is summarized in Fig. 2. Besides proinflammatory cytokines, some growth factors, such as heparin-binding EGF-like growth factor, EGF, TGF-α, insulin-like growth factor-1, and FGF-2, play a role in the proliferative process during epithelialization [1, 31].

There are some other signaling pathways that contribute to epithelialization. For example, autocrine Wnt/β-catenin signaling controls the differentiation and self-renewal of iSCs [41]. The differentiation of iSCs also depends on Notch signaling, and Notch1/2/3 receptors and Jagged 1 are expressed in the mouse supra-basal epidermis, whereas Jagged 2 is expressed in the basal layer cells [42].

## hSCs and sSCs exert plasticity in epithelialization

SCs from skin appendages, including hSCs and sSCs, contribute to the self-regeneration of appendages and epithelialization in wound healing. The hSCs are relatively comprehensive according to their complexity and diversity. Distinct markers reflect different locations and actions of hSCs. Mostly, hSCs reside in the permanent non-cyclic follicle portion (bulges), and they express specific markers, such as CD34; keratin15/19 (K15/19); leucine-rich-repeat-containing G protein-coupled receptor 5 (LGR5); SRY-box 9 (SOX9); LIM homeobox 2 (LHX2); nuclear factor of activated T cells, cytoplasmic 1 (NFATC1); T-box 1 (TBX1); and transcription factor 3 (TCF3). Besides, hSCs reside in the infundibulum (upper part of the isthmus), and they express LRIG1. The hSCs also reside in the isthmus (the junctions between the hair follicles and the sebaceous gland), and they express LRIG1, LGR6, BLIMP1, and PLET1 (Fig. 1) [6, 28, 30]. Usually, sSCs express LRIG1, LGR6, and BLIMP1 [6, 30]. The duct SCs reside at the opening of the gland, and they express GATA-binding protein6 (GATA6) (Fig. 1). These SCs contribute to interfollicular epithelialization in wound healing [16].

During wound healing, hSCs migrate upwards to the interfollicular epidermis. However, different populations of hSCs may have opposite effects. For example, the SCs expressing CD34, LRIG1, and K15 contribute to healing of the interfollicular epidermis in a rapid but temporary manner. In contrast, the LGR5-, SOX9-, and GLI1-expressing SCs remain in the interfollicular epidermis for a longer time even in the post-wounding stage [30, 43]. Wound healing tends to be faster in skin with higher hair density (e.g., the fully covered scalp). A chronic wound heals quickly when treated with skin grafts containing hair follicles [44]. In addition, the rate of wound healing correlates with synchronized hair follicle cycling in mice because wound healing accelerates during the anagen phase of hair follicle cycling, which has different epithelial, endothelial, and inflammatory cell types [45].

Proinflammatory cytokines, including IL-1, IL-17, and TNF, promote hair follicle neogenesis and epithelialization in wound healing. IL-1 and IL-7 can expand the population of active γδT cells, which subsequently enhance the proliferation and mobilization of hSCs [32]. Recently, it was reported that Treg cells participate in the migration and differentiation of Lgr5-positive hSCs in epithelialization by activating the CXCL5-IL-17 inflammatory axis [46]. TNF-α is crucial in the macrophage-induced hair follicle telogen-anagen transition, and it participates in hair follicle neogenesis in wounds. TNF-α treatment increases β-catenin levels in a PI3K/AKT-dependent manner in Lgr5-positive hSCs, which are important for hair follicle neogenesis [38].

The Wnt signaling pathway mediates the proliferation of hSCs and the epithelialization process in tissue regeneration [47]. The epithelial-mesenchymal interaction is instrumental in hair follicle morphogenesis, and it involves the activation of Wnt, bone morphogenetic protein, Shh, Notch, TGF-β, and platelet-derived growth factor signaling [48].

## Therapeutic strategies targeting epidermal SCs and relevant regulators

Epidermal SCs are promising in the treatment of skin wounds. SCs have long been explored in therapeutic epidermal autografts [49], which can be derived from unpurified epidermal cell cultures containing both iSCs and hSCs. Direct spray harboring SCs have replaced passaged epidermal keratinocytes as this preferred method of burn therapy accelerates wound healing with less scarring [6]. However, only small and superficial wounds are suitable for spray therapy. Topical application and injection of hSCs have been conducted in both rat models and ulcer patients, and it showed better wound closure by reducing inflammation and improving epithelialization and angiogenesis [50–52]. The administration of SCs not only accelerates wound healing, but also enhances the physiological function of skin. Besides epidermal SCs, other SCs applicable in wound healing include mesenchymal SCs, adipose-derived SCs, endothelial progenitor cells, and umbilical cord perivascular cells [53]. Wounds treated with mesenchymal SCs show significantly less inflammatory cells and proinflammatory cytokines. Umbilical cord-matrix SCs increase M2 macrophages in diabetic wounds, which further upregulate the secretion of IL-10 and VEFG but downregulate the production of IL-6 and TNF-α [54]. Mesenchymal SCs also reduce scar formation by secreting a variety of anti-fibrotic cytokines, including hepatocyte growth factor, IL-10, and adrenomedullin [53, 55]. Adipose-derived SCs and induced pluripotent SCs reduce scar formation in mouse experiments [56, 57].

Proinflammatory cytokines initially play a beneficial role in acute wound healing by promoting the proliferation and antimicrobial peptide production of keratinocytes. However, overproduction of proinflammatory cytokines may lead to prolonged inflammation and wound healing. Therefore, blocking excessive proinflammatory cytokines exerts a therapeutic effect in chronic wound healing. Patients with chronic wounds have higher systemic and local levels of TNF-α. Topical application of anti-TNF-α neutralizing antibodies blunts leukocyte recruitment and NF-κB activation, alters the balance between M1 and M2 macrophages, enhances matrix synthesis, and finally accelerates wound healing in the secretory leukocyte protease inhibitor (SLPI) null mouse model, which has age-related delayed human wound healing [58]. Topical application of infliximab and adalimumab, monoclonal antibodies of anti-TNF, is effective for patients with chronic ulcers [59, 60].

Blocking IL-1β activity using a neutralizing antibody suppresses the proinflammatory factors (IL-1β, MMP-9, TNF-α, and IL-6), but it enhances the healing-associated markers (CD206, insulin-like growth factor-1, TGF-β, and IL-10) in macrophages from diabetic patients or a murine model [61]. Also, neutralizing anti-IL-1β antibody or IL-1R antagonist upregulates the pro-wound healing phenotype of macrophages and improves healing in diabetic mice [61, 62].

Anti-IL-17A antibodies strengthen re-epithelialization of wounds in obese diabetic mice by altering the proportion of M1/M2 macrophage populations without any effect on scarring or fibrosis [63]. Local application of recombinant IL-17A leads to delayed wound healing and accelerated neutrophil accumulation in mice [64]. Subcutaneous injection of recombinant mouse IL-17 enhances macrophage infiltration in mice treated with full-thickness excision, accompanied by aggravated fibrogenesis, delayed wound healing, and amplified inflammation [65]. Recent therapies involving SCs and proinflammatory cytokines are summarized in Table 1.

**Table 1:** Therapies involving SCs and proinflammatory cytokines

Targeted cells or factors	Molecule or cell tested	Route of application	Subject	Therapeutic effect	Ref
Stem cells	Hair follicle stem cells	Direct application-hair skin graft	Patients	Less ulcer area, more granulation tissue formation and vascularization, and better innervation of the wound bed	[50, 51]
	Hair follicle stem cells	Intradermal injection	Rats	Less inflammation, more granulation tissue formation, and faster vascularization and epithelialization	[52]
	Hair follicle stem cells	Local injection	Patients	Increased hair density and hair follicle number	[66, 67]
	Mesenchymal stem cells	Injection/spray	Mice/patients	Less inflammatory cells, proinflammatory cytokines, and scar formation as well as faster wound closure	[55]
TNF-α	Anti-TNF-α neutralizing antibody	Topical application	Mice/patients	Less leukocyte recruitment, rebalance of M1/M2 macrophages, more matrix synthesis, and faster wound healing	[58–60]
IL-1	Anti-IL-1β neutralizing antibody	Topical application	Cultured macrophages/mice	Lower proinflammatory macrophage phenotype and proinflammatory cytokines expression. Faster re-epithelialization and granulation tissue formation and more collagen deposition	[61]
	IL-1 receptor antagonist	Topical application	Mice	Less leukocyte and macrophage recruitment and faster wound healing	[62]
IL-17	Anti-IL-17A antibody	Local injection	Mice	More pro-healing macrophages and better wound closure	[63]

## Clinical application of stem cell- or growth factor-related therapies

With the progress of SC application in skin wound healing, regeneration of hair follicles begins to attract more interest in functional skin construction or hair loss diseases. Adipose tissue-derived hSCs display an improvement in hair density visually and an expansion in the number of hair follicles in patients with androgenic alopecia [66]. Along with SCs, platelet-rich plasma, being a main resource of growth factors, improves cell proliferation, differentiation, and angiogenesis and results in wound healing and hair follicle regeneration [67]. Platelet-rich plasma contains at least six major growth factors, including platelet-derived growth factor, EGF, FGF, TGF-β, VEGF, and insulin-like growth factor-1, which are important in tissue regeneration. Besides, platelet-rich plasma contains proinflammatory cytokines including the IL family and TNF-α family, which contributes to tissue regeneration [68, 69]. In addition, platelet-rich plasma combined with hyaluronic acid or fat grafting improves the epithelialization in patients of chronic ulcers and regeneration of soft tissue defects [68, 70].

Different populations of SCs and growth factors have been applied in multiple tissue regeneration to meet both therapeutic and esthetic needs. Adipose-derived SCs, containing stromal vascular fraction, can improve dermal elasticity by increasing collagen and elastin synthesis and remodeling facial scars [71]. Stromal vascular fraction cells can also be used in breast reconstruction and oncoplastic surgery, for it improves vascularization and the fibrogenic activity of fibroblasts and further benefits adipose tissue survival and 3D organization [72]. Therefore, the effective application of SCs combined with growth factors or novel biomaterials diminishes unnecessary injuries, leading to a new level in the regeneration medicine.

## Conclusions and prospective views

Since skin wound healing remains an intractable problem in clinics, an increasing number of researches have been conducted to explain the mechanisms involved in the healing process where SCs play an important role. Abundant factors, especially proinflammatory cytokines, regulate and participate in SC migration, proliferation, and differentiation in wound healing and hair follicle neogenesis as well as show a therapeutic effect via distinct signaling pathways. Rebalance of these cytokines probably benefits the transition from the inflammation phase to the proliferation phase in skin wounds and improves the healing process. The therapeutic strategies associated with SCs or proinflammatory cytokines have achieved success to some extent. However, few strategies can efficiently reverse the deficiencies that contribute to chronic wounds and restore the tissue to its pre-injured state. Some side effects, such as scar formation, abnormal pigmentation, and tumorigenesis, still hamper the development of ideal treatments. Despite unrealistic commercial and clinical expectations, tissue-engineered skin based on SCs has delivered considerable benefits to patients with burns and chronic wounds. Combination of inflammatory cytokines or growth factors with SCs in an appropriate dosage and timeline seems promising in wound healing therapies. Further investigation of interactions between immune cells and SCs is necessary to elucidate the mechanisms of wound healing and will help make progress in clinical applications. It is likely that with the increasing knowledge of SCs and tissue engineering, better therapies with less side effects and financial costs will be developed in the near future.

## Data Availability

Not applicable.

## Abbreviations

EGF: Epidermal growth factor
FGF: Fibroblast growth factor
Fn14: Fibroblast growth factor-inducible 14
IL: Interleukin
MMP: Matrix metalloproteinase
NF-κB: Nuclear factor-κB
PI3K: Phosphatidylinositide 3-kinase
SC: Stem cell
hSC: Hair follicle SC
iSC: Interfollicular epidermal SC
sSC: Sebaceous gland SC
TGF: Transforming growth factor
TNF: Tumor necrosis factor
TWEAK: TNF-like weak inducer of apoptosis
